# Streptococcal taxonomy based on genome sequence analyses

**DOI:** 10.12688/f1000research.2-67.v1

**Published:** 2013-03-01

**Authors:** Cristiane C Thompson, Vanessa E Emmel, Erica L Fonseca, Michel A Marin, Ana Carolina P Vicente

**Affiliations:** 1 Laboratory of Molecular Genetics of Microorganisms, Oswaldo Cruz Institute (IOC - FIOCRUZ) Avenida Brasil 4365, Manguinhos, Rio de Janeiro, P. O. Box 926, Zip Code 21040-360, Brazil

## Abstract

The identification of the clinically relevant viridans streptococci group, at species level, is still problematic. The aim of this study was to extract taxonomic information from the complete genome sequences of 67 streptococci, comprising 19 species, by means of genomic analyses, multilocus sequence analysis (MLSA), average amino acid identity (AAI), genomic signatures, genome-to-genome distances (GGD) and codon usage bias. We then attempted to determine the usefulness of these genomic tools for species identification in streptococci. Our results showed that MLSA, AAI and GGD analyses are robust markers to identify streptococci at the species level, for instance, *S. pneumoniae*, *S. mitis*, and *S. oralis*. A *Streptococcus* species can be defined as a group of strains that share ≥ 95% DNA similarity in MLSA and AAI, and > 70% DNA identity in GGD. This approach allows an advanced understanding of bacterial diversity.

## Introduction

Bacteria are subjected to numerous forces driving their diversification. As a consequence, different strains of a single bacterial species sometimes have the ability to explore distinct niches, to be pathogenic or non-pathogenic and to present different metabolic pathways ^1, 2^. In such a scenario, the identification of bacteria isolates to the species level is a hard task ^1, 2^.

Currently, the genus *Streptococcus* comprises 99 recognized species, many of which are associated with disease in humans and animals ( http://www.bacterio.net/s/streptococcus.html). The viridans group streptococci (VGS) encompass four phylogenetic clusters: Mitis, Mutans, Salivarius and Anginosus, which are part of the human microbiota, being isolated mainly from the oral cavity, gastrointestinal and genitourinary tracts ^3^. The Mitis group currently includes the important pathogen *S. pneumoniae* and 12 other recognized species, *S. australis*, *S. cristatus* (formerly *S. crista*), *S. gordonii*, *S. infantis*, *S.mitis*, *S. oligofermentans*, *S. oralis*, *S. parasanguinis* (formerly *S. parasanguis*), *S. peroris*, *S. pseudopneumoniae*, *S. sanguinis* (formerly *S. sanguis*) and *S. sinensis*. The Anginosus group includes three recognized species, *S. anginosus*, *S. constellatus* (including two subspecies *S. constellatus* subsp. *constellatus* and *S. constellatus pharyngis*) and *S. intermedius*, and the Salivarius group includes *S. salivarius*, *S. vestibularis*, and *S. thermophilus*.

Currently, bacterial species are considered to be a group of strains (including the type strain) that are characterized by a certain degree of phenotypic consistency, showing > 70% DNA-DNA hybridization values and over 97% 16S rRNA sequence similarity ^4, 5^. Identification of streptococci is based on the current taxonomic standards using a combination of 16S rRNA gene sequence analyses, DNA-DNA hybridization, serologic and phenotypic data; however, they have been strikingly resistant to satisfactory classification, reflected in frequently changing nomenclature ^6, 7^. For instance, the 16S rRNA gene sequences of *S. mitis* and *S. oralis* are almost identical (> 99%) to *S. pneumoniae*, making the use of this information alone insufficient to distinguish these species ^8^.

Recent studies have used whole genome analysis to determine the taxonomic relationships among bacterial species ^9– 14^. In order to determine the robustness of genomic markers in streptococci species delineation, we analyzed a collection of 67 complete genomes. The availability of whole genome sequences of several closely related species, for instance, *S. mitis - S. oralis - S. pneumoniae*, and *S. salivarius - S. thermophilus - S. vestibularis*, formed an ideal test case for the establishment of the genomic taxonomy of streptococci.

## Material and methods

### Genome sequence data

The genomic sequences of 67 streptococci that were publicly available for download by June 2 ^nd^, 2011 at the National Center for Biotechnology Information (NCBI) under the project accession number indicated in Table 1 were used in this study. The following analyses were performed according to Thompson *et al.* (2009) ^13^ and are briefly described below.

**Table 1.** Genomic features of the streptococci. G+C content (%): guanine + cytosine content (%). No. of CDs: number of coding DNA sequence. *Nc*: effective number of codons.

Organism	GenBank accession no.	Genome size (nt)	G+C content (%)	No. of CDS	*Nc*
***S. agalactiae* A909**	CP000114	2,127,839	35	1996	44.9
***S. agalactiae* NEM316**	AL732656	2,211,485	35	2094	45.2
***S. agalactiae* 2603VR**	AE009948	2,160,267	35	2124	45.1
***S. anginosus* F0211**	AECT00000000	1,993,709	38	2035	50.6
***S. bovis* ATCC 700338**	AEEL00000000	2,050,893	37	2088	44.5
***S. downei* F0415**	AEKN00000000	2,239,421	43	2204	54.4
***S. dysgalactiae* subsp. *equisimilis* GGS-124**	AP010935	2,106,340	39	2094	50.3
***S. equi* subsp. *equi* 4047**	FM204883	2,253,793	41	2001	52.6
***S. equi* subsp. *zooepidemicus***	FM204884	2,149,868	41	1869	52.4
***S. equi* subsp. *zooepidemicus* MGCS10565**	CP001129	2,024,171	41	1893	52.3
***S. gallolyticus* subsp. *gallolyticus* TX20005**	AEEM00000000	2,214,091	37	2218	44.5
***S. gallolyticus* UCN34**	FN597254	2,350,911	37	2223	44.4
***S. gordonii* str. *Challis* substr. CH1**	CP000725	2,196,662	40	2051	52.4
***S. infantis* SK1302**	AEDY00000000	1,792,252	39	2102	48.9
***S. infantarius* subsp. *infantarius* ATCC BAA-102**	ABJK00000000	1,925,087	37	2051	44.0
***S. mitis* B6**	FN568063	2,146,611	39	2004	50.4
***S. mitis* SK321**	AEDT00000000	1,873,702	40	1757	49.8
***S. mutans* NN2025**	AP010655	2,013,587	36	1895	46.4
***S. mutans* UA159**	AE014133	2,030,921	36	1960	46.5
***S. oralis* ATCC 35037**	AEDW00000000	1,884,712	41	1793	51.4
***S. parasanguinis* ATCC 15912**	ADVN00000000	2,124,730	41	2035	52.8
***S. parasanguinis* F0405**	AEKM00000000	2,050,302	41	1978	52.9
***S. pneumoniae* AP200**	CP002121	2,130,580	39	2216	50.3
***S. pneumoniae* ATCC 700669**	FM211187	2,221,315	39	1990	50.0
***S. pneumoniae* CGSP14**	CP001033	2,209,198	39	2206	50.3
***S. pneumoniae* D39**	CP000410	2,046,115	39	1914	49.8
***S. pneumoniae* G54**	CP001015	2,078,953	39	2114	50.0
***S. pneumoniae* Hungary19A-6**	CP000936	2,245,615	39	2155	50.2
***S. pneumoniae* INV104**	FQ312030	2,142,122	39	1824	49.9
***S. pneumoniae* INV200**	FQ312029	2,093,317	39	1930	50.0
***S. pneumoniae* JJA**	CP000919	2,120,234	39	2123	50.2
***S. pneumoniae* OXC141**	FQ312027	2,036,867	39	1824	49.9
***S. pneumoniae* P1031**	CP000920	2,111,882	39	2073	50.1
***S. pneumoniae* R6**	AE007317	2,038,615	39	2042	50.1
***S. pneumoniae* Taiwan19F-14**	CP000921	2,112,148	39	2044	50.1
***S. pneumoniae* TCH843119A**	CP001993	2,088,772	39	2275	50.4
***S. pneumoniae* TIGR4**	AE005672	2,160,842	39	2105	50.0
***S. pneumoniae* 670-6B**	CP002176	2,240,045	39	2352	50.4
***S. pneumoniae* 70585**	CP000918	2,184,682	39	2202	50.1
***S. pseudoporcinus* SPIN 20026**	AENS00000000	2,111,372	36	2030	48.6
***S. pyogenes* MGAS315**	AE014074	1,900,521	38	1865	49.1
***S. pyogenes* MGAS2096**	CP000261	1,860,355	38	1898	49.4
***S. pyogenes* MGAS5005**	CP000017	1,838,554	38	1865	48.9
***S. pyogenes* MGAS6180**	CP000056	1,897,573	38	1894	48.9
***S. pyogenes* MGAS8232**	AE009949	1,895,017	38	1839	49.0
***S. pyogenes* MGAS9429**	CP000259	1,836,467	38	1877	49.0
***S. pyogenes* MGAS10270**	CP000260	1,928,252	38	1986	49.0
***S. pyogenes* MGAS10394**	CP000003	1,899,877	38	1886	49.2
***S. pyogenes* MGAS10750**	CP000262	1,937,111	38	1979	49.1
***S. pyogenes* M1 GAS**	AE004092	1,852,441	38	1696	48.8
***S. pyogenes* NZ131**	CP000829	1,815,785	38	1700	48.8
***S. pyogenes* SSI-1**	BA000034	1,894,275	38	1859	49.1
***S. pyogenes* str. *Manfredo***	AM295007	1,841,271	38	1745	48.9
***S. salivarius* SK126**	ACLO00000000	2,128,332	40	1992	47.0
***S. sanguinis* ATCC 49296**	AEPO00000000	2,054,852	41	2013	51.7
***S. sanguinis* SK36**	CP000387	2,388,435	43	2270	54.5
***S. sanguinis* VMC66**	AEVH00000000	2,311,949	43	2260	54.5
***S. suis* BM407**	FM252032	2,146,229	41	1932	52.0
***S. suis* GZ1**	CP000837	2,038,034	41	1979	52.4
***S. suis* P17**	AM946016	2,007,491	41	1824	51.9
***S. suis* SC84**	FM252031	2,095,898	41	1898	52.0
***S. thermophilus* CNRZ1066**	CP000024	1,796,226	39	1915	47.0
***S. thermophilus* LMD-9**	CP000419	1,856,368	39	1709	46.8
***S. thermophilus* LMG 18311**	CP000023	1,796,846	39	1888	46.9
***S. thermophilus* ND03**	CP002340	1,831,949	39	1919	46.8
***S. uberis* 0140J**	AM946015	1,852,352	36	1762	46.4
***S. vestibularis* F0396**	AEKO00000000	2,022,289	39	1979	47.1

### 16S rRNA gene sequence analysis and multilocus sequence analysis (MLSA)

The 16S rRNA gene sequences and the gene sequences used for MLSA were obtained from GenBank ( http://www.ncbi.nlm.nih.gov). The MLSA approach was based on the concatenated sequences of five house-keeping genes ( *aroE*, *ddl*, *gki*, *pheS* and *recA*) ^15, 16^. The concatenated sequences were aligned with ClustalX program ^17^. The phylogenetic inference was based on the neighbour-joining genetic distance method (NJ) ^18^ using MEGA5 ^19^. Distance estimations were obtained according to the Kimura-2-parameter ^20^ for 16S rRNA gene and MLSA. The reliability of each tree topology was checked by 2000 bootstrap replications ^21^.

### Average amino acid identity (AAI)

The AAI of all conserved protein-coding genes was calculated as described previously ^22^. Conserved protein-coding genes between a pair of genomes were determined by whole-genome pairwise sequence comparisons using the BLASTp algorithm ^23^. For these comparisons, all protein-coding sequences (CDSs) from one genome were searched against the genomic sequence of the other genome. The genetic relatedness between a pair of genomes was measured by the AAI of all conserved genes between the two genomes as computed by the BLAST algorithm. By this approach, a value of < 95% AAI of protein-coding genes indicates separate species.

### Codon usage

Codon usage bias was calculated for each genome. The effective number of codons used in a sequence ( *Nc*) ^24^ was calculated using CHIPS ( http://emboss.bioinformatics.nl/cgi-bin/emboss/chips) with the default parameters.

### Determination of dinucleotide relative abundance values and genomic dissimilarity

Mononucleotide and dinucleotide frequencies were calculated using COMPSEQ ( http://emboss.bioinformatics.nl/cgi-bin/emboss/compseq) with default parameters. Dinucleotide relative abundances (ρ*XY) were calculated using the equation ρ*XY = fXY/fXfY where fXY denotes the frequency of dinucleotide XY, and fX and fY denote the frequencies of X and Y, respectively. The difference in genome signature between two sequences is expressed by the genomic dissimilarity (δ*), which is the average absolute dinucleotide of relative abundance difference between two sequences, and were calculated using the equation: δ*(f,g) = 1/16Σ|ρ*XY (f) - ρ*XY (g)| (multiplied by 1000 for convenience), where the sum extends over all dinucleotides ^25^.

### Genome-to-genome distances (GGD)

The genome distance was calculated using genome-to-genome distance calculator (GGDC) ^26^. Distances between a pair of genomes were determined by whole-genome pairwise sequence comparisons using BLAST ^23^. For these comparisons, algorithms were used to determine high-scoring segment pairs (HSPs) for inferring intergenomic distances for species delimitation. The corresponding distance threshold can be used for species delimitation ^26^.

## Results and discussion

In this work we compared complete genomes for 67 streptococci comprising 19 species to address their taxonomic position. A previous study with a small set of streptococci genomes (eight) and species (four), using a combination of several genomic analyses, showed the applicability of this approach in streptococci taxonomy ^9^. Overall our analysis, using a large data set, showed that genomic taxonomy is an accurate approach to clearly define the streptococci species. The taxonomic resolution of the 16S rRNA, AAI, MLSA, GGD and codon usage analysis for streptococci species definition is summarized in Table 2.

**Table 2.** Taxonomic resolution of genomic analyses of streptococci species. MLSA: multilocus sequence analysis. AAI: amino acid identity. GGD: genome to genome distance. *Nc*: effective number of codons.

	16S rRNA (%)	MLSA (%)	AAI (%)	GGD (%)	Codon usage ( *Nc*)
**Intraspecies**	≥99	≥95	≥95	>70	-
*S. pyogenes*	≥99	≥98	>97	>70	49
*S. agalactiae*	99	100	98	>70	45
*S. equi*	99	98	>96	>70	52
*S. suis*	100	100	100	>70	52
*S. pneumoniae*	99	≥97	>97	>70	50
*S.thermophilus*	99	100	>97	>70	47
**Interspecies**	≤99	<95	<95	<70	44-54
*S. thermophilus-salivarius-vestibularis*	99	<94	<92	<70	47
*S. pneumoniae-mitis-oralis*	>99	<94	<93	<70	50–51

Raw data: MLSA nucleotide sequences Similarity values for the MLSA nucleotide sequences (lower left) and 16S rRNA gene sequences (upper right) for the Streptococcus strains.

Raw data: average amino acid identiy Percentage of average amino acid identity (AAI) between Streptococcus strains.

Raw data: genomic signatures Genomic dissimilarity [δ(f,g)] values between Streptococcus strains.

### General genomic features

The complete genome of the streptococci comprised a single chromosome. The estimated size of the genomes ranged from 1.7 Mb ( *S. infantis*) to 2.3 Mb ( *S. sanguinis*). The number of CDS varied from 1,700 ( *S. pyogenes*) to 2,352 ( *S. pneumoniae*) (Table 1). The average G+C content of streptococci genomes ranged from 35% to 43%. These species presented a variable interspecies genome size and G+C content, indicating heterogeneity within the genus *Streptococcus*. One of the reasons for this variability could be associated with the frequent occurrence of horizontal gene transfer events ^27– 29^.

### Phylogenetic reconstructions by 16S rRNA and MLSA

**Figure 1.**
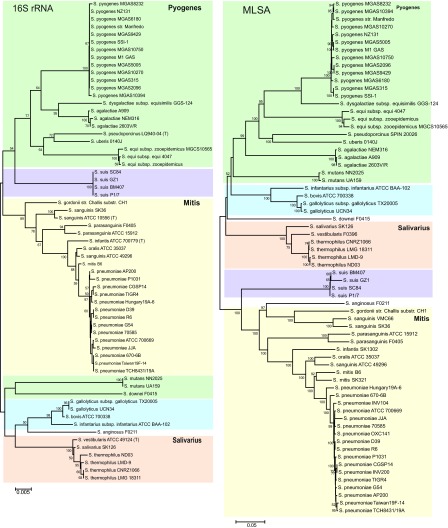
Neighbor-joining tree based on 16S rRNA gene sequences and MLSA concatenated sequences of *Streptococcus*. The numbers at the nodes indicate the values of bootstrap statistics after 2000 replications, and values below 50% are not shown. Bars, 0.005% and 0.02% estimated sequence divergence.

MLSA and 16S rRNA phylogenetic trees showed similar topologies (Figure 1). The MLSA was performed using five instead of the seven genes applied in the pneumococcus multilocus sequence typing (MLST) scheme ( http://spneumoniae.mlst.net/) ^15, 16^. Three genes, *aroE*, *ddl* and *gki*, are from the MLST scheme, and *pheS* and *rec*A were included in this work. The concatenation of these genes (7741 bp) allowed an accurate delineation of the streptococci species considered here. The nucleotide sequence similarities were much lower for MLSA than 16S rRNA gene. A pairwise comparison of MLSA among the species revealed sequence similarity between 67% and 100%, while the 16S rRNA gene sequence similarities varied from 92% to 100%. At the intraspecies level, the similarity values ranged from 95% to 100% for MLSA, and 99% to 100% for the 16S rRNA gene sequences. The closest species within the Mitis ( *S. pneumoniae* - *S. oralis* - *S. mitis*) and Salivarius groups ( *S. vestibulares - S. salivarius - S. thermophilus*) were clearly placed apart from each other by MLSA, while these species had almost identical 16S rRNA gene sequences (≥ 99% sequence similarity). A previously study showed that *recA* analysis is a valuable tool for proper identification of pneumococci in routine diagnostics, but limitations on discrimination of other members of the Mitis group were observed ^30^. *S. sanguinis* ATCC 49296 showed a much closer relationship with *S. oralis* ATCC 35037T (95% similarity) than to other *S. sanguinis* strains (77% similarity), suggesting it belongs to the species *S. oralis*. In addition, *S. bovis* ATCC 700338 was placed in the *S. gallolyticus* cluster with 98% MLSA sequence similarity. This work showed that MLSA, using this new combination of five concatenated genes ( *aro*E, *ddl*, *gki*, *phe*S and *rec*A), distinct from the *Streptococcus* MLST scheme, allowed a proper identification of most streptococci species, even within the VGS group.

### Average amino acid identity (AAI)

The percentage of average amino acid identity (AAI) among streptococci species ranges from 68% to 94%, while within species it varies from 95% to 100%. The VGS species *S. pneumoniae*, *S. mitis* and *S. oralis* shared 89–93% AAI. The species *S. salivarius*, *S. thermophilus* and *S. vestibularis* showed a maximum AAI of 93%. *S. sanguinis* ATCC 49296 and *S. oralis* ATCC 35037 showed 96% identity and *S. bovis* ATCC 700338 and *S. gallolyticus* strains had 98% identity. These findings suggest that strains ATCC 49296 and ATCC 700338 belong to the species *S. oralis* and *S. gallolyticus*, respectively. According to our analyses the AAI and MLSA are the most useful genomic features for the elucidation of streptococci taxonomy.

### Genome signature

The genomic dissimilarity values among streptococci were between 3 and 127, while the intraspecies values were between 0 and 17. Streptococci within the VGS group, for instance, *S. salivarius*, *S. thermophilus* and *S. vestibularis* species, showed dissimilarity values between 5 and 12 and *S. pneumoniae, S. mitis* and *S. oralis* species had dissimilarity values between 3 and 14. Thus, there was not a clear differentiation of these closely related species within the VGS group on the basis of the genomic dissimilarity values. This could be due to the extensive recombination and horizontal gene transfer events which occur between closely related streptococci species that share ecological niches ^12, 30^.

On the other hand, species within the Pyogenic group had a distinct genomic signature, with values ranging from 13 to 85. However, genome signatures alone have significant limitations when used as phylogenetic markers for differentiating members of the VGS. The exact mechanisms that generate and maintain the genome signatures are complex, but possibly involve differences in species-specific compositional bias, i.e., G+C content, G+C and A+T skews, codon bias, and mutation bias ^32, 33^.

### Codon usage bias ( *Nc*)

*Nc* values provide a meaningful measure of the extent of codon preference in a genome, values range between 20 (extremely biased genome where one codon is used per amino acid) and 61 (all synonymous codons are used). Within the set of 67 complete streptococci genomes examined in this study, the *Nc* ranged from 44.0 to 54.5 (Table 1). For instance, *S. pneumoniae* - *S. oralis* - *S. mitis* species had *Nc* values of 50, 51 and 50, respectively. The Salivarius group ( *S. vestibulares - S. salivarius - S. thermophilus*), and *S. bovis* ATCC 700338 *- S. gallolyticus* showed *Nc* values of 47 and 44.5, respectively. Overall, codon usage bias was very similar among the streptococci species investigated. However, *S. sanguinis* ATCC 49296 showed a much closer *Nc* value with the *S. oralis* ATCC 35037 (51.7 and 51.4, respectively) than other *S. sanguinis* strains (54.5), which was in agreement with the other analyses used in this study.

### Genome distance analysis

The GGD was calculated only for closely related species that were not differentiated by 16S rRNA gene sequence analysis (Figure 1). Based on GGD analysis the species within the Mitis and Salivarius groups were identified as separate species, showing GGD values analogous to the < 70% discriminatory value used for DNA-DNA hybridization. Conversely, *S. bovis* ATCC 700338 and *S. gallolyticus* were identified as belonging to the same species by GGD.

*S. bovis* ATCC 700338 (biotype II) and *S. gallolyticus* as well as *S. sanguinis* ATCC 49296 and *S. oralis* ATCC 35037T were not separated and, therefore, according to this analysis would be classified as the same species, respectively. It was shown that *S. bovis* biotype I and II/2 isolates were, in fact, *S. gallolyticus* ^34^, and *S. sanguinis* ATCC 49296 was placed into *S. oralis* species by GGD analysis. A misidentification of *S. sanguinis* ATCC 49296 has already been shown by means of biochemical and serological properties by Narikawa and colleagues ^35^.

Another interesting result is that the *S. parasanguinis* ATCC 15912 and F0405 strains were found to be at the upper limits for definition as members of the same species based on different genomic analyses. For instance, they shared 95% AAI, 94% identity by MLSA, a value of 17 on the basis of genomic signature and < 70% similarity in GGD. Therefore, based on these genomic markers, these *S. parasanguinis* strains could, in fact, be separate species. This data reflects the complexity of bacterial species delineation, since these organisms are all under a constant evolutionary process.

## Conclusion

The delineation of closely related streptococci species was evident in this genomic study. Different methods produced different levels of taxonomic resolution. The methods with the higher resolution for species identification were MLSA and AAI, while closely related species had similar *Nc* values and genomic signatures. Based on the genomic analyses, a *Streptococcus* species can be defined as a group of strains that shares ≥ 95% identity in MLSA and AAI, and > 70% identity in GGD. This definition may be useful to advance the taxonomy of *Streptococcus*. This approach allows an advanced understanding of bacterial diversity and identification.

## References

[1] Gevers D Cohan FM Lawrence JG: Opinion: Re-evaluating prokaryotic species. *Nat Rev Microbiol.* 2005;3(9):733–9 10.1038/nrmicro1236 16138101

[2] Cohan FM Koeppel AF: The origins of ecological diversity in prokaryotes. *Curr Biol.* 2008;18(21):R1024–34 10.1016/j.cub.2008.09.014 19000803

[3] Alam S Brailsford SR Whiley RA: PCR-Based methods for genotyping viridans group streptococci. *J Clin Microbiol.* 1999;37(9):2772–6 10449450 10.1128/jcm.37.9.2772-2776.1999 PMC85375

[4] Stackebrandt E Goebel BM: Taxonomic Note: A place for DNA-DNA reassociation and 16S ribosomal-RNA sequence analysis in the present species definition in bacteriology. *Int J Syst Bacteriol.* 1994;44(4):846–849 10.1099/00207713-44-4-846

[5] Wayne LG Brenner DJ Colwell RR: Report of the ad hoc committee on reconciliation of approaches to bacterial systematics. *Int J Syst Bacteriol.* 1987;37(4):463–464 10.1099/00207713-37-4-463

[6] Hoshino T Fujiwara T Kilian M: Use of phylogenetic and phenotypic analyses to identify nonhemolytic streptococci isolated from bacteremic patients. *J Clin Microbiol.* 2005;43(12):6073–85 10.1128/JCM.43.12.6073-6085.2005 16333101 PMC1317212

[7] Kawamura Y Hou XG Sultana F: Determination of 16S rRNA sequences of Streptococcus mitis and Streptococcus gordonii and phylogenetic relationships among members of the genus Streptococcus. *Int J Syst Bacteriol.* 1995;45(2):406–8 10.1099/00207713-45-2-406 7537076

[8] Suzuki N Seki M Nakano Y: Discrimination of Streptococcus pneumoniae from viridans group streptococci by genomic subtractive hybridization. *J Clin Microbiol.* 2005;43(9):4528–34 10.1128/JCM.43.9.4528-4534.2005 16145102 PMC1234109

[9] Coenye T Vandamme P: Extracting phylogenetic information from whole-genome sequencing projects: the lactic acid bacteria as a test case. *Microbiology.* 2003;149(pt 12):3507–17 10.1099/mic.0.26515-0 14663083

[10] Coenye T Gevers D Van de Peer Y: Towards a prokaryotic genomic taxonomy. *FEMS Microbiol Rev.* 2005;29(2):147–67 10.1016/j.femsre.2004.11.004 15808739

[11] Richter SS Heilmann KP Dohrn CL: Accuracy of phenotypic methods for identification of Streptococcus pneumoniae isolates included in surveillance programs. *J Clin Microbiol.* 2008;46(7):2184–8 10.1128/JCM.00461-08 18495854 PMC2446902

[12] Croucher NJ Harris SR Fraser C: Rapid pneumococcal evolution in response to clinical interventions. *Science.* 2011;331(6016):430–4 10.1126/science.1198545 21273480 PMC3648787

[13] Thompson CC Vicente ACP Souza RC: Genomic taxonomy of Vibrios. *BMC Evol Biol.* 2009;9:258 10.1186/1471-2148-9-258 19860885 PMC2777879

[14] Thompson CC Vieira NM Vicente AC: Towards a genome based taxonomy of Mycoplasmas. *Infect Genet Evol.* 2011;11(7):1798–804 10.1016/j.meegid.2011.07.020 21840423

[15] Enright MC Spratt BG: A multilocus sequence typing scheme for Streptococcus pneumoniae: identification of clones associated with serious invasive disease. *Microbiology.* 1998;144(Pt 11):3049–60 10.1099/00221287-144-11-3049 9846740

[16] Hanage WP Fraser C Spratt BG: Sequences, sequence clusters and bacterial species. *Philos Trans R Soc Lond B Biol Sci.* 2006;361(1475):1917–27 10.1098/rstb.2006.1917 17062411 PMC1764932

[17] Thompson JD Gibson TJ Plewniak F: The CLUSTAL_X windows interface: flexible strategies for multiple sequence alignment aided by quality analysis tools. *Nucleic Acids Res.* 1997;25(24):4876–82 10.1093/nar/25.24.4876 9396791 PMC147148

[18] Saitou N Nei M: The neighbor-joining method: a new method for reconstructing phylogenetic trees. *Mol Biol Evol.* 1987;4(4):406–25 3447015 10.1093/oxfordjournals.molbev.a040454

[19] Tamura K Peterson D Peterson N: MEGA5: molecular evolutionary genetics analysis using maximum likelihood, evolutionary distance, and maximum parsimony methods. *Mol Biol Evol.* 2011;28(10):2731–9 10.1093/molbev/msr121 21546353 PMC3203626

[20] Kimura M: A simple method for estimating evolutionary rates of base substitutions through comparative studies of nucleotide sequences. *J Mol Evol.* 1980;16(2):111–120 10.1007/BF01731581 7463489

[21] Felsenstein J: Confidence Limits on Phylogenies: An Approach Using the Bootstrap. *Evolution.* 1985;39(4):783–791 10.2307/2408678 28561359

[22] Konstantinidis KT Tiedje JM: Towards a genome-based taxonomy for prokaryotes. *J Bacteriol.* 2005;187(18):6258–64 10.1128/JB.187.18.6258-6264.2005 16159757 PMC1236649

[23] Altschul SF Madden TL Schäffer AA: Gapped BLAST and PSI-BLAST: a new generation of protein database search programs. *Nucleic Acids Res.* 1997;25(17):3389–402 10.1093/nar/25.17.3389 9254694 PMC146917

[24] Wright F: The 'effective number of codons' used in a gene. *Gene.* 1990;87(1):23–9 10.1016/0378-1119(90)90491-9 2110097

[25] Karlin S Mrázek J Campbell AM: Compositional biases of bacterial genomes and evolutionary implications. *J Bacteriol.* 1997;179(12):3899–913 9190805 10.1128/jb.179.12.3899-3913.1997 PMC179198

[26] Auch AF Klenk HP Göker M: Standard operating procedure for calculating genome-to-genome distances based on high-scoring segment pairs. *Stand Genomic Sci.* 2010;2(1):142–8 10.4056/sigs.541628 21304686 PMC3035261

[27] Zhang A Yang M Hu P: Comparative genomic analysis of Streptococcus suis reveals significant genomic diversity among different serotypes. *BMC genomics.* 2011;12:523 10.1186/1471-2164-12-523 22026465 PMC3227697

[28] Bellanger X Roberts AP Morel C: Conjugative transfer of the integrative conjugative elements ICESt1 and ICESt3 from Streptococcus thermophilus. *J Bacteriol.* 2009;191(8):2764–75 10.1128/JB.01412-08 19181800 PMC2668402

[29] Harvey RM Stroeher UH Ogunniyi AD: A variable region within the genome of Streptococcus pneumoniae contributes to strain-strain variation in virulence. *PloS One.* 2011;6(5):e19650 10.1371/journal.pone.0019650 21573186 PMC3088708

[30] Zbinden A Köhler N Bloemberg GV: recA-based PCR assay for accurate differentiation of Streptococcus pneumoniae from other viridans streptococci. *J Clin Microbiol.* 2011;49(2):523–7 10.1128/JCM.01450-10 21147955 PMC3043496

[31] Donati C Hiller NL Tettelin H: Structure and dynamics of the pan-genome of Streptococcus pneumoniae and closely related species. *Genome Biol.* 2010;11(10):R107 10.1186/gb-2010-11-10-r107 21034474 PMC3218663

[32] Karlin S: Global dinucleotide signatures and analysis of genomic heterogeneity. *Curr Opin Microbiol.* 1998;1(15):598–610 10.1016/S1369-5274(98)80095-7 10066522

[33] Foerstner KU von Mering C Hooper SD: Environments shape the nucleotide composition of genomes. *EMBO Rep.* 2005;6(12):1208–13 10.1038/sj.embor.7400538 16200051 PMC1369203

[34] Devriese LA Vandamme P Pot B: Differentiation between Streptococcus gallolyticus strains of human clinical and veterinary origins and Streptococcus bovis strains from the intestinal tracts of ruminants. *J Clin Microbiol.* 1998;36(12):3520–3 9817865 10.1128/jcm.36.12.3520-3523.1998 PMC105232

[35] Narikawa S Suzuki Y Takahashi M: Streptococcus oralis previously identified as uncommon "Streptococcus sanguis" in Behçet’s disease. *Arch Oral Biol.* 1995;40(8):685–90 10.1016/0003-9969(95)00042-N 7487566

