# Supplementary material for: Streptococcal taxonomy based on genome sequence analyses
Source: F1000Res. 2013 Mar 1;2:67. [Version 1] doi: 10.12688/f1000research.2-67.v1 (PMC3799547; doi:10.12688/f1000research.2-67.v1)
Supplement: Peer review history

# Peer review: Referee response for version 1

**Published:** 2013-04-03

**Authors:** Bruno Gomez-Gil

**Affiliations:** 1 CIAD, A.C., Mazatlán Unit for Aquaculture and Environmental Management, Mazatlán, Mexico

The article is well written with an appropriate title and abstract. The methods are adequate for the aims of the study, but I would suggest that including the Average Nucleotide Identity (ANI) analysis as suggested by Rosello-Mora *et al*. 2006, would certainly improve the manuscript. The online analysis can be found here http://www.imedea.uib.es/jspecies/index.html.The conclusions are adequate and the data sufficient to replicate all the analyses.The data are openly accessible at GenBank.

I have read this submission. I believe that I have an appropriate level of expertise to confirm that it is of an acceptable scientific standard.

# Peer review: Referee response for version 1

**Published:** 2013-04-03

**Authors:** Tomoo Sawabe

**Affiliations:** 1 Laboratory of Microbiology, Graduate School of Fisheries Sciences, Hokkaido University, Hakodate, Japan

- Title and abstract are good enough to attract readers in the scientific community.
- Genome based multi-gene sequence comparison is one of the promising tools to analyse bacterial populations. To achieve the analysis for *Streptococcus*, the authors carefully designed massive data genome analysis. The results are strong enough and supported by the results of the analysis.
- The conclusion is clear that authors proposed a threshold value on the basis of genome-based MLSA in *Streptococcus* bacteria.

I have read this submission. I believe that I have an appropriate level of expertise to confirm that it is of an acceptable scientific standard.
